# Functional Polymer and Packaging Technology for Bakery Products

**DOI:** 10.3390/polym14183793

**Published:** 2022-09-10

**Authors:** Horman San, Yeyen Laorenza, Ehsan Behzadfar, Uruchaya Sonchaeng, Kiattichai Wadaugsorn, Janenutch Sodsai, Thitiporn Kaewpetch, Khwanchat Promhuad, Atcharawan Srisa, Phanwipa Wongphan, Nathdanai Harnkarnsujarit

**Affiliations:** 1 Department of Packaging and Materials Technology, Faculty of Agro-Industry, Kasetsart University, 50 Ngam Wong Wan Rd., Latyao, Chatuchak, Bangkok 10900, Thailand; 2 Chemical Engineering Department, Ryerson University, Toronto, ON M5B 2K3, Canada; 3 Sustainable Packaging Lab, School of Graphic Communications Management, Ryerson University, Toronto, ON M5B 2K3, Canada; 4 Center for Advanced Studies for Agriculture and Food, Kasetsart University, 50 Ngam Wong Wan Rd., Latyao, Chatuchak, Bangkok 10900, Thailand

**Keywords:** functional polymer, active packaging technology, bakery products, antimicrobial, shelf life

## Abstract

Polymeric materials including plastic and paper are commonly used as packaging for bakery products. The incorporation of active substances produces functional polymers that can effectively retain the quality and safety of packaged products. Polymeric materials can be used to produce a variety of package forms such as film, tray, pouch, rigid container and multilayer film. This review summarizes recent findings and developments of functional polymeric packaging for bakery products. Functional polymerics are mainly produced by the incorporation of non-volatile and volatile active substances that effectively retain the quality of packaged bakery products. Antimicrobial agents (either synthetic or natural substances) have been intensively investigated, whereas advances in coating technology with functional materials either as edible coatings or non-edible coatings have also preserved the quality of packaged bakery products. Recent patents demonstrate novel structural packaging designs combined with active functions to extend the shelf life of bakery products. Other forms of active packaging technology for bakery products include oxygen absorbers and ethanol emitters. The latest research progress of functional polymeric packaging for bakery products, which provides important reference value for reducing the waste and improving the quality of packaged products, is demonstrated. Moreover, the review systematically analyzed the spoilage factors of baked products from physicochemical, chemical and microbiological perspectives. Functional packaging using polymeric materials can be used to preserve the quality of packaged bakery products.

## 1. Introduction

The new market research report “Baking Ingredients Market by Type, Application, and Region—Forecast to 2026”, published by Newswire [1], forecasts that the global baking ingredients market will grow from USD 16.6 billion in 2021 to USD 22.3 billion by 2026, at a Compound Annual Growth Rate (CAGR) of 6.0%. Globally, one-third of the food produced for human consumption is lost as waste, amounting to 1.3 billion tons per year and worth USD 1 trillion. According to Goryńska-Goldmann et al. (2021) [2], bakery companies’ average daily losses ranged from 9.7% to 14.4% of the production volume, including bread and fresh pastry losses. Many variables, such as nutritional value, taste, freshness, shelf life and consumer attractiveness, impact the quality of bakery products that have a short shelf life, resulting from adverse changes that begin immediately after baking and cause worsening sensory features and crumb texture. Aging of bread is manifested by partial decrease in humidity, growth of filamentous fungi and yeasts, and staling [3].

Active packaging systems are designed to “deliberately incorporate components that would release or absorb substances into or from the packaged food or the environment surrounding the food.” Active packaging materials are intended to extend the shelf life or to maintain or improve the condition of packaged food [4,5]. Garcia and Copetti (2016) [6] distinguished two kinds of antimicrobial packaging. Firstly, the antimicrobial agent migrates from the package to the product’s surface, whereas secondly, it is in direct contact with the food. Various factors influence the release of antimicrobial chemicals from an edible film, such as electrostatic interactions between the antimicrobial agent and the polymer chains, osmosis, and environmentally induced changes in structure. Jideani and Vogt (2016) [7] evaluated the antimicrobial properties of active packaging systems used to prolong the shelf life of bread. Integrating synthetic or organic antimicrobial agents has become one of the active packaging strategies used to protect food goods against deterioration and microbiological growth [8]. Organic acids and their salts (sodium benzoate, potassium sorbate), sulfites, nitrites, antibiotics, and alcohols are typical synthetic antimicrobial chemicals, whereas natural antimicrobial substances such as organic acids, bacteriocins (nisin and lacticin), grape seed extracts, herbal extracts, and enzymes (peroxidase and lysozyme) have also been shown to inhibit the growth of microorganisms.

Some essential oils, such as extracted plant-based compounds, have known antimicrobial, antifungal, antioxidant and anti-carcinogenic properties [7,9]. Nanditha and Prabhasankar (2009) [10] stated that bakery foods most often contain fats and oils. These progressively oxidize during storage, resulting in rancidity and deterioration in the sensory attributes of the food products. Oxidation inhibitors or antioxidants can be used to prevent the autooxidation of fats and oils in packaged foods. Tertiary butyl hydroxy quinone (TBHQ), gallates, butylated hydroxy anisole (BHA), butylated hydroxytoluene (BHT) and other synthetic antioxidants are frequently utilized in bakery products. These compounds are highly effective in preventing the oxidation of fat, are available at a low cost and are easy to incorporate into food products. To improve shelf life and consumer acceptance, several natural antioxidants including α-tocopherol, β-carotene and ascorbic acid can be used in place of synthetic antioxidants. By contrast, several plant extracts from fruits and vegetables contain significant amounts of substances that are functional antioxidants, such as apple and strawberries (vitamin C, bioflavonoids, chalcones), banana fruit (gallocatechin), carrot (α-and β-carotene, phenolic compounds), grapes (phenols, catechins), citrus fruits (β-crytoxanthin, bioflavonoids, chalcones, vitamin C) and garlic, onion, and leeks (allicin, flavonoids, vitamin C, selenium, sulfur) [10,11,12,13]. Antioxidants are also evident in various foodstuffs including milk, cocoa, seaweeds, vanilla, garcinia and agro-industrial wastes but they have yet to be used in bread goods [10]. Similarly, Gavahian et al. 2020 [14] discovered that certain natural essential oils act as preservatives for baked products by inhibiting microbial growth. Both conventional and innovative alternative extraction techniques are used to extract essential oils, and some have antimicrobial properties. For instance, extracted essential oils from orange peel contain limonene, with terpenoids from thymol and carvacrol, and phenylpropenes from eugenol and vanillin and other herbal essential oils. These antimicrobial compounds are effective against spoiling microbial growth in bakery products. Essential oils can also be used as natural preservatives and antimicrobial agents in bakery product packaging.

Kotsianis et al. (2002) [15] reported a review on the use of modified atmosphere packaging (MAP) technology in bakery products. Packaging materials for MAP should have low gas permeability, sealability characteristics, clarity, and antifog properties. They should also have excellent barrier properties to prevent gases from leaking. MAP machinery also needs consideration because it offers benefits by minimizing product costs and extending the shelf life of baked goods. Alternative MAP techniques include oxygen scavengers, which remove oxygen from packaging to prevent aerobic growth of pathogenic bacteria. Ethanol can also be added to the package headspace to prevent the growth of mold, yeast, lactobacilli, and other microbials. Both of these alternative MAP methods can be applied as sachets. Galic et al. (2009) [16] found that the storage conditions and packaging systems of baked goods affected their shelf life. The optimal packaging material must be considered based on each type of baked product by taking into consideration variables such as gases and water vapor permeability, durability, usage, and functionality. For instance, low-density polypropylene (PE-LD) had higher gas permeability, thereby extending bread crumb crispiness. MAP also plays a significant role in delaying the deterioration of bakery goods. Differing packaging materials have various microbiological and intrinsic qualities that influence preservation and bakery product shelf life [17].

Previous reviews examined extending the shelf life of bread using various innovative packaging technologies for microbial growth control in bread and bakery products [6,7,10,14,16]. Garcia and Copetti [6] demonstrated alternative and holistic methods to improve the shelf life of bakery products including controlling unit operations in processing, adding natural preservatives, and utilization of predictive methods. Jideani and Vogt [7] highlighted the roles of antimicrobial packaging produced by the double extrusion process to achieve homogeneous blends between antimicrobial and polymers, whereas the potential combination of antimicrobial agents (allyl isothiocyanate and potassium sorbate) was incorporated into polymers as natural antimicrobial protection for bread. Moreover, the effects of natural and synthetic antioxidants used in bakery products for increasing shelf life have been reviewed [10,14]. Galic et al. [16] reviewed applications of chemical kinetic concepts to food quality deteriorations which efficiently determined shelf-life of bakery, whereas packaging technology had a significant role in product quality. Incorporations of active substances into polymers produces functional polymeric packaging which subsequently affects quality of packaged bakery. These functional polymers include either paper or plastic and can release active functions, e.g., antioxidant and antimicrobial, which delay quality deteriorations of packaged products.

This review assessed the up-to-date information on new packaging innovations for bakery products, such as polymeric functional plastics that incorporate volatile and/or non-volatile compounds, edible polymeric coating, functional paper and coating technology, and other various forms of active packaging for bakery products such as oxygen absorbers and ethanol emitters. This review on functional packaging systems and materials can be used to address additional issues and initiatives for maintaining and enhancing product quality by preserving the nutritional and organoleptic qualities of bakery goods and shelf life. Additionally, it discusses the role of functional packaging in increasing consumer trust and reducing the negative environmental impacts of packaging waste and food loss. The review also provides future research directions for packaging and material technology, food and bakery product development, and food business strategy.

## 2. Bakery Deterioration Factors through Packaging System

Products from bakeries are prone to spoilage through microbiological, chemical, and physical deterioration. The quality criteria of all types of bread have to be established to determine whether they are acceptable to the consumer or not. Commonly used characteristics related to bread quality are loaf volume, crumb color, and crumb aesthetic. The consumer may squeeze the loaf to determine the softness and make a quick judgment of the freshness. The choice of ingredients, formulations, equipment, and processing methods can affect final product quality. The roles of ingredients and methods of mixing have a large impact on the shelf life of bread. Staling is one of the main processes that causes bread to lose quality [18], whereas package permeabilities including water vapor permeability (WVP) and oxygen permeability (OP) also have major impacts on quality preservation of packaged bakery, examples of which are shown in Table 1.

### 2.1. Physico-Chemical and Chemical Deteriorations

Baked goods are flour-based foods baked in an oven such as bread, cookies, cakes, donuts, pasties, and pies [19]. They can undergo a variety of changes during storing that reduce their freshness. The deterioration processes affect the properties of both crumb and crust aesthetics, due to loss of moisture and migration of moisture between the two. The bread becomes firmer over time due to changes in the degree of crystallinity of the starch fraction. Recrystallization of starch also causes flavor and aroma loss. Sandwich bread will last anywhere from 3–5 days, whereas craft breads typically last less than a day. Products that can remain soft and mold-free for up to 12 days are now available to consumers [18,20].

Rancidity is a chemical deterioration induced by lipid oxidation of bakery components. Fat rancidity is brought on by storage time and low water activity [21]. Oxygen is a major factor in accelerating lipid oxidation. Packaging with lower oxygen permeability tends to prevent lipid oxidation; however, bakery products commonly have high porosity with high amounts of residual oxygen embedded in food matrices. This oxygen should also be removed from the package and, therefore, oxygen scavenging packaging is important to eliminate oxygen levels after sealing [22]. In items with water activity of under 0.3, this issue typically arises very quickly. Oxidation reduces until it rises once again after the product water activity reaches 0.5 [23]. In addition to the fats used with baked products, other ingredients with naturally high fat content can act as sources of free radicals including oat products and nuts. Some lipases are most active with high water activities but they will still react at lower water activities (0.25), creating challenges [20].

**Figure 1:**
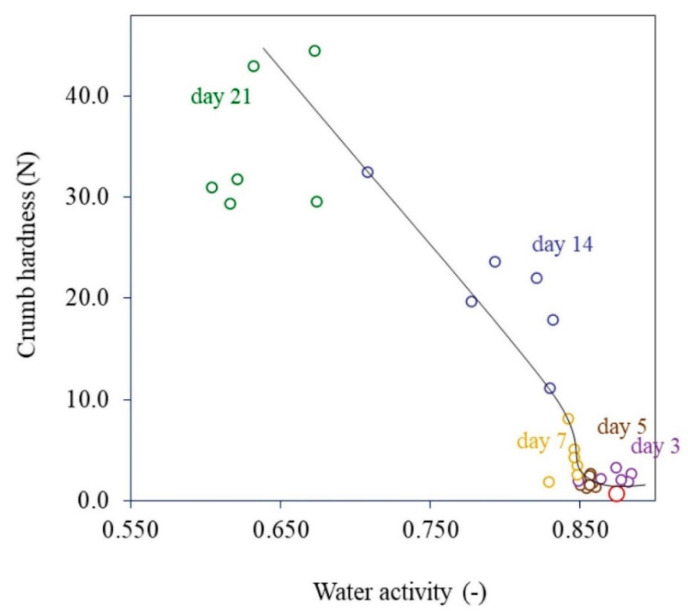
The relationship between water activity and crumb hardness during storage for 21 days in biodegradable films at room temperature (Reproduced with permission from Srisa and Harnkarnsujarit (2020) [24]).

Retrogradation of starch components leads to staling of bakery products which contributes to loss of water and freshness. Water strongly influences the textural properties of bakery products. Higher moisture contents give a softer texture, whereas lower moisture causes harder textural properties. Organoleptic characteristics and physical appearance undergo significant changes over time, associated with the movement of water from both inside and outside the product matrix. The difference in partial pressure of water vapor between the inside of the package and the surrounding environment is the driving force for water transport across the packaging material. For particular, the influence of water vapor permeation might make sandwich bread crumbs firmer or soften cookies while they are being stored [20]. Bakery product quality also depends on packaging and packaging material. The permeability of packaging materials can influence moisture migration by affecting the relative humidity of the atmosphere surrounding the product. Packaging materials with low moisture vapor transmission rates create high relative humidity in the package atmosphere, and this means that equilibrium can be reached between the product and the atmosphere [20]. The hardening of breadcrumbs occurs because of moisture loss due to retrogradation of the starch components and evaporation of water. Water transport is driven by the difference in relative vapor pressure between the bread and the environment, which subsequently reduces water activity. Srisa and Harnkarnsujarit (2020) [24] found a relationship between water activity and hardening in breadcrumbs during storage for 21 days in biodegradable films (Figure 1). They reported that the hardness significantly increased with reduced water activity crumb value. Increasing the degree of recrystallization increased hardness and reduced water activity during storage. Increased crumb hardness followed a zero-order rate equation (R^2^ = 0.85 to 0.98), with the rate dependent on release of volatile *trans*-cinnamaldehyde, which further interacted with bread components, including lipids [24,25].

**Figure 2:**
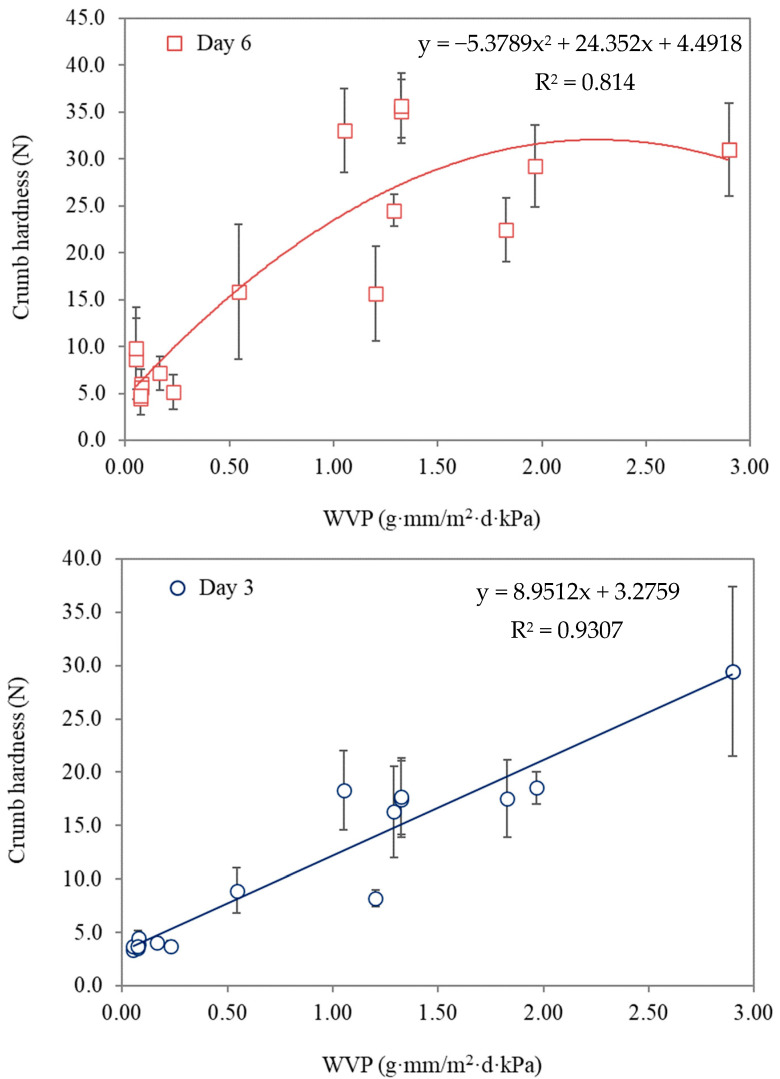
The relationship between water vapor permeability and crumb hardness at 3 and 6 days of storage at room temperature (reproduced with permission from Bumbudsanpharoke et al. (2022) [26]).

Food packaging permeability plays a key role in the hardening of packaged bread. Films with higher water vapor permeability allow for a higher transfer of water vapor which causes a higher driving force for moisture transportation. Bumbudsanpharoke et al. (2022) [26] found a linear relationship between water vapor permeability of biodegradable and non-biodegradable plastic blend films (PBAT, PBS and LLDPE) and hardness of bread crumb stored for 3 days, whereas a non-linear relationship was found after 6 days of storage (Figure 2). Increasing water vapor permeability (WVP) gave higher permeation rates, which subsequently increased crumb hardness due to enhancing the water transfer from crumbs. Higher water vapor permeability commonly facilitated water transfer from higher relative vapor pressure (RVP) in bread crumbs to lower RVP in the surroundings, leading to decreased water activity [26,27].

### 2.2. Microbial Deteriorations

Microbiological spoilage is a major problem for bakery products, often limiting their shelf life. Microbial growth causes economic loss for both manufacturers and consumers as a result of poor packaging, sanitation practices in manufacturing, storage conditions and low product turnover [28]. Microorganism can be found in raw materials of the baking goods which contaminated during processing and packaging, or immediately after the oven. Fungal deterioration impacts wheat-based bakery products as *Penicillium*, *Aspergillus*, *Cladosporium* and *Neurospora*, whereas *Bacillus subtilis* and *Bacillus licheniformis* deterioration are less common [29,30]. Water activity affects bread products, and mold development and other microbial spoilage are the most common problems. Mold spoilage is a serious and costly problem for bakeries. Normal cooking temperatures destroy fungal spores, and post-process contamination from airborne spores and contact with contaminated surfaces must be prevented. Filamentous fungi involved in bread spoilage include *Rhizopus* sp., and *Mucor* sp., *Penicillium* sp., *Eurotium* sp., *Aspergillus* sp. and *Monilia sitophilia*. *Rhizopus stolonifer*, also referred to as the “bread mold”, is one of the most prevalent. Mold growth appears when bread is stored in low-humidity environments, with generation of mycotoxins also as a concern. *Eurotium* species are the first fungi to colonize contaminated water, followed by *Penicillium* and *Aspergillus*. Losses of bakery goods due to mold spoilage range from 1 to 5%, depending on the weather, the type of product, and the processing techniques. The most common fungi related to losses in bakery products belong to the genera *Aspergillus, Cladosporium*, *Endomyces*, *Fusarium*, *Monilia*, *Mucor*, *Penicillium*, and *Rhizopus* [31]. Yeast problems also occur in bakery products. Wild yeasts include *Trichosporon variable*, *Saccharomyces*, *Pichia*, and Zygosaccharomyces. *Saccharomyces* sp. produces white spots in bread, leading to the term chalk bread [28].

Furthermore, rope spoilage in bread is caused by bacteria from the genus *Bacillus* and occurs in the summer when the climate is most conducive to bacterial growth which is related to temperature changing. The bacteria involved include *Bacillus subtilis*, *Bacillus licheniformis*, *Bacillus magaterium* and *Bacillus cereus* [28]. Meanwhile, bacteria possibly contaminate baked goods, and *Bacillus* is a major contaminant from raw ingredients. *Staphylococcus aureus* is one type of bacteria known to contaminate pie filling and can cause food poisoning outbreaks from cream-filled bakery products [28]. Before the development of visible indications of deterioration, Needham et al. (2005) [32] examined the ability to distinguish between microbiological spoilage brought on by bacteria, yeast and fungi and enzymic spoilage brought on by lipoxygenase after 48 h using cluster analysis. The volatiles released by the various types of bread spoilage and unspoiled bread analogs were determined using gas chromatography–mass spectrometry. The amounts of each microorganism increased over time, according to microbial analyses. Mold growth is the major microbial spoilage agent for many baked products. Generally, mold spores in fresh bread and other baked goods are killed by the baking process. Bread spoilage caused by mold typically occurs after baking by contamination by the air, bakery surfaces, equipment, food handlers, or raw ingredients [28,33].

Permeability of polymeric packaging potentially causes the deterioration of bakery products. Additionally, oxygen permeation allows the growth of aerobic microorganisms such as mold in bakery products. Higher oxygen permeability commonly accelerates aerobic fungal growth. The reduction in oxygen levels in the package limits the mold growth in bakery products [34,35]. On the other hand, water vapor permeability also plays a key role in microbial growth at intermediate water activity levels. Moisture loss from packaged products reduces water activity and partially inhibits microbial growth. Bumbudsanpharoke et al. (2022) [26] indicated that mold growth in packaged bread had insignificant correlation with oxygen permeability, whereas moisture loss from bread crumbs due to high water vapor permeability showed a dominant effect on bacterial growth due to decreasing water activity.

**Table 1:** Functional polymers and packaging technology for bakery products.

Functional Packaging	Active Agents	Packaging Form	Type of Bakery	Remarkable Results	References
Non-volatile active compounds	Zinc oxide nanoparticles	Chitosan-carboxymethyl cellulose film	Preservative-free soft sliced wheat bread	➢ Coated films had decreased water vapor permeability, maintained higher moisture content, and increased water activity than the control ➢ ZnO 1% and 2% inhibited *Aspergillus niger* and no mold growth on the bread for 15 days	[36]
	Natamycin	Chitosan-natamycin vacuum packaged and spraying	Phyllo pastry	➢ Chitosan and natamycin preserved sensory attributes for 17 days at 4 °C storage and inhibited Enterococci and *Clostridium* spp. up to 18 days	[37]
	Sodium propionate	Polypropylene-sodium propionate film	Bread	➢ Enhanced mechanical and thermal stability, increased hydrophilicity ➢ Films showed antimicrobial activity against both Gram-negative and Gram-positive microbials, and bread showed less spoilage by mold on day 7 during storage	[38]
	Silver nanoparticles	Polyvinyl chloride film	Sliced Bread	➢ Ag-nanoparticles 1% inhibited microorganisms in bread for 15 days of storage at 26 °C ➢ Improved the properties of PVC material	[39]
	ε-poly-L-lysine (ε-PL)	Starch film	Bread	➢ Inhibition against *A. parasiticus* and *P. expansum* and diminished aflatoxin by more than 93.90% after 7 days of testing	[40]
	ZnO nanoparticles	Gelatin- polyethylene film	Sponge cake	➢ Prevented fungal growth for 28 days and maintained cake chemical and organoleptic quality	[41]
	TiO_2_	Potato starch film	Sliced bread	➢ 1% TiO_2_ coating increased water vapor barrier properties and inhibited the growth of *Bacillus subtilis* and *Escherichia coli*	[42]
	Chitosan	Chitosan-PLA film	Sliced bread	➢ All modified chitosan nanoparticles (CSNPs) showed capacity to inhibit *S. aureus* as high as > 98%, improved elongation at break and maintained oxygen permeation ability in a standard range for food packaging	[43]
	Sulfur quantum dot	Alginate film	Bread	➢ Integrated film improved tensile strength by 18%, UV barrier by 82% and antioxidant activity, while maintaining stiffness and WVP; sulfur-based compounds had antibacterial action against *L. monocytogenes* and *E. coli*, as well as against fungi such *A. niger* and *P. chrysogenum* and delayed the appearance of mold on bread for 14 days	[44]
	Sorbate anion	Polypropylene bag	White bread	➢ The coated film retained organoleptic characteristics, moisture analysis, peroxide evolution and mold count on bread for up to 12 days at ambient temperature and inhibited growth of *Escherichia coli*, *Pseudomonas aeruginosa*, *Salmonella enterica subsp*. Arizona, Staphylococcus aureus and *Campylobacter jejuni*	[45]
Volatile active compounds	Cinnamaldehyde	Gliadin films	Sliced bread	➢ Highly effective against fungal growth for both in vitro and food packing systems; cinnamaldehyde volatility from the solution forming film inhibited activity of *P. expansum* and *A. niger* over 10 days	[25]
	Oregano essential oil	Nonwoven tissue/polypropylene-based sachet	Preservative-free sliced bread	➢ Inhibited the growth of *E. coli*, *Salmonella* Enteritidis and *Penicillium* sp., bread texture increased with storage time, but sachets had no effect; higher OEO concentration imparted unpleasant sensory effects (bitter taste and strong odor)	[46]
	Apricot kernel essential oil	Chitosan film	Sliced bread	➢ The blended film decreased WVP, lower solubility and moisture content enhanced tensile strength and scavenging activity for both H_2_O_2_ and DPPH ➢ Delayed bacterial growth as *Bacillus subtilis* and *Escherichia coli* protected against fungal growth of sliced bread within the packaging on day 10	[47]
	Grapefruit seed extract/Chitosan	Poly(ε-caprolactone)/chitosan film	Preservative-free bread	➢ Grapefruit seed extract incorporation led to increased pits on the film surface but there was no mold growth on packaged bread with film containing ≥ 1.0 mL/g grapefruit seed extract after 7 days	[48]
	*trans*-cinnamaldehyde	PLA/PBAT film	Bread	➢ Increased *trans*-cinnamaldehyde contributed to reduced barrier properties and decreased mechanical properties due to plasticization and pores embedded in films ➢ Films with *trans*-cinnamaldehyde from 2% and above effectively inhibited the microbial growth of bacteria and fungi for more than 21 days at 30 °C	[24]
	Eugenol and citral	Corn starch microcapsule sachet	Sliced bread	➢ The EOs-containing sachets were effective in inhibiting the growth of molds and yeasts in media and sliced bread without affecting the sensory properties of bread	[49]
	Thymol	PLA/PBSA film	Preservative-free bread	➢ Effective against fungal growth up to 9 days and improved thermal and barrier properties as well as decreased glass transition temperature, melting temperature and crystallinity ➢ Thymol decreased the permeability of water vapor, oxygen and carbon dioxide, tensile strength and Young’s modulus but increased elongation at break	[50]
	Sorbitol/Grapefruit seed extract	Corn starch-chitosan film	Bread	➢ Inhibition against *A. niger* and extended bread shelf life up to 20 days at 25 °C and 59% RH ➢ Had low moisture content, water vapor permeability, solubility, high tensile strength and high antifungal activity	[51]
	*Cymbopogon citratus* essential oil	Cashew gum-gelatin film	Bread	➢ The incorporated film extended shelf life to 6 days compared with the control at only 3 days	[52]
	Carvacrol	PLA/PBAT film	Preservative-free bread	➢ PLA/PBAT blend ratio controlled the strength, permeability and release behavior of carvacrol ➢ Film showed delayed fungal growth and sporulation of *Penicillium* sp. and *Rhizopus* sp. with 2.0–2.3 times increased shelf life	[53]
	Cinnamon oil	Natural rubber pressure-sensitive adhesive patch	Banana cake	➢ NR-PSA/CO patch delayed the growth of bacterial and fungal strains as *Escherichia coli*, *Staphylococcus aureus*, *Aspergillus niger* with extension of the 4-day shelf life	[54]
	*Piper betel* Linn extract	Poly (vinyl alcohol) film	Sliced bread	➢ Films had high UV blocking and antimicrobial efficiency ➢ Inhibition against bacteria such as *E. coli*, *S. typhimurium*, *S. aureus* and *P. aeruginosa* with 3% of extract concentration and preserved bread quality for 45 days at room temperature	[55]
	Cinnamaldehyde Limonene Eugenol	Fish gelatin-based nanofiber mat	Bread	➢ The incorporated mat had radical scavenging activity, ferric reducing antioxidant power and better encapsulation with the electrospinning method ➢ Inhibited the growth of *E. coli*, *S. aureus* and *A. niger* ➢ There was no fungal spot on bread antimicrobial packing	[56]
	Thyme essential oil	Poly (3-hydroxybutyrate-co-4-hydroxybutyrate) film	White bread	➢ Films containing 30% *v*/*w* of thyme essential oils extended the shelf life of bread up to 5 days depending on visible mold growth observation ➢ Films enhanced both water vapor permeability and elongation at break	[57]
	Schiff base	PLA film	Bread	➢ Delayed growth of fungi on bread slices to day 5 compared with the control at day 3 ➢ Films also killed the bacteria plasma membrane as an inhibition zone	[58]
Functional paper and paperboard	PLA	Coated paperboard	-	➢ PLA-coated paperboards improved water barrier properties through decreasing water vapor permeability and increase in water contact angle	[59]
	Vanillin with dimethyl sulfoxide, ethyl alcohol, and chitosan	Coated paper	-	➢ Each coating successfully inhibited growth of bacteria; however, efficiency varied depending on mixture concentration	[60]
	Wax	Coated paper	Milk cake	➢ Maintained sensory acceptability up to 21 days because the coated paper minimized moisture loss from milk cake	[61]
	Cinnamon essential oil	Coated paper	-	➢ Significantly reduced mold growth by direct migration in packaging and demonstrated resistance to *Rhizopusstolonifer* growth at 4% concentration	[62]
	Ag/TiO_2_-SiO_2_, Ag/N-TiO_2_, or Au/TiO_2_	Paper modification	“Pave” bread	➢ Characteristics of the paper including busting, tensile, tearing and breaking resistance decreased as the composite content increased. ➢ Increased whiteness of the paper ➢ Ag/TiO_2_-SiO_2_-paper and Ag/N-TiO_2_-paper extended bread shelf life by more than 2 days compared to unmodified paper in both ambient and refrigeration conditions by offering an efficient control on acidity and yeast and mold growth; Au/TiO_2_ had no influence on shelf-life extension indicating that nano-Ag had preservation activity and photoactivity	[63]
	Chitosan	Coating paper	-	➢ Coating increased the glossiness of paper as the chitosan filled surface porosity and improved moisture resistance, mechanical characteristics and flexibility	[64]
	TiO_2_ Ag-TiO_2_ Ag-TiO_2_-zeolite	Bleached paper	Bread	➢ Improved barrier properties such as air permeability, water vapor permeability and reduced grease permeation ➢ Bread packed in Ag-TiO_2_ paper had an extended shelf life for 2 more days than the control package based on yeast and mold growth	[65]
	Nano-carbon	Wrapping paper	Brownie cake	➢ Activated carbon-modified bamboo wrapping paper preserved nutrients in food and specifically reduced the level of microbial contamination on brownie cake	[66]
	Blending of alginate, carboxymethyl cellulose, carrageenan, and grapefruit seed extract	Coated paper	Mined fish cake	➢ The biopolymer coating improved water and grease resistance, surface hydrophobicity and tensile properties of paper ➢ Coated paper showed strong antimicrobial activity against *L. monocytogenes* and *E. coli*	[67]
	Chitosan/Ag/TiO_2_	Coated paper	Clarified butter	➢ Coated paper had better opacity values, reduced water vapor and oxygen permeabilities and decreased oil permeability ➢ Inhibition against E. coli at 70.36% on an agar plate and 73.28% in butter samples, as well as against yeasts and molds at 77.02% on an agar plate and 79.28% in butter samples ➢ After six months, the peroxide value increased 6.47-fold with P-CH-Ag/TiO_2_ compared to uncoated at 36.71-fold	[68]
	Starch, NaCl, Aquaseal	Paper bag	Bread	➢ Relative humidity (RH) of sandwich paper rose to 72% and enhanced bread sensory quality and freshness up to 72 h of storage, extending the shelf life	[69]
	Geraniol	Paper sachet	Sliced bread	➢ PBS/geraniol-10% exhibited inhibition against *Escherichia coli* and *Bacillus cereus* with degradation of white bread with total plate count, yeasts, and mold count on day 42 with an antimicrobial sachet, whereas no fungus was spotted on white bread surface preserved with an antimicrobial sachet for the entire 63-day test period	[70]
	Schiff base PLA	Kraft paper coating	Bread	➢ Paper properties showed increased smoothness, maintained heat-sealing strength, decreased air porosity value and higher oil-grease resistance	[58]
Edible and non-edible coating	*Lactobacillus acidophilus*	Edible starch/probiotic coating	Bread	➢ Probiotic coating technique obtained microencapsulation of *Lactobacillus acidophilus* and starch-based material coated onto surface of baked breads resulting in better protection on bread crust and sensory acceptability	[71]
	Ag/TiO_2_ nanocomposite	HDPE film	White bread	➢ Bread stored in Ag/TiO_2_-based packaging inhibited proliferation of yeast/molds, *Bacillus cereus* and *Bacillus subtilis* due to scavenging more water and oxygen molecules in the packaging headspace	[72]
	Potassium sorbate and citric acid	Potato starch, inverted sugar, sucrose coating solution	Mini panettones	➢ Panettones with an edible coating containing both additives showed fungal growth from 40 days, and with 1 g/kg potassium sorbate only, yeast and mold growth were not detected until 48 days ➢ During storage, there was reduced water activity, moisture, elasticity and cohesiveness of panettones with additives, whereas the reverse occurred in the controls	[73]
	Triticale flour	Edible coating and spraying	Muffin	➢ Triticale film coating worked well to prolong the staling process, keeping the fresh muffins softer during 10 days of storage because of delaying crumb-firming kinetics	[74]
	Star anise essential oil and thymol	PP/SAEO/PET/TH/LDPE film	Preservative-free sliced wheat bread	➢ Insect repellent activity sustained the bread for up to 23 days and prevented antimicrobial growth for 14 days; the developed film had low tensile strength and elastic modulus	[75]
	Garlic extract and Bread aroma	Coating on PE film	Preservative-free sliced pan loaf	➢ PE film coated with zein containing 0.5% garlic extract and bread aroma maintained bread free of mold growth for 30 days	[76]
	Lactic acid bacteria	Edible lactic acid bacteria coating	Wheat bread	➢ Coating with *Streptococcus salivarius* subsp. *thermophilus, Lactobacillus delbrueckii* subsp. *Bulgaricus, Lactobacillus acidophilus,* sodium alginate, whey and glycerol had the best protective properties against microbial spoilage ➢ Incorporation of lactic acid bacteria in a coating containing alginate ensured good viability for 120 h ➢ Coating diminished *A. niger* and *P. chrysogenum* in wheat bread	[77]
	Okra mucilage	Edible okra mucilage gum surface coating	Biscuit	➢ Coated biscuits were preserved from deterioration and microbial spoilage with improved moisture barrier quality	[78]
Absorber/ Emitter	Iron-based oxygen absorber	Sachet (FreshPax^®^)	Cracker	➢ Prevented oxidation and extended the shelf life of military ration crackers packaged in hermetically sealed tin cans for 44 weeks	[79]
	Oxygen absorber and ethanol emitter	Sachet	Wheat bread	➢ Ethanol emitter increased the shelf life of bread by up to 24 days based on sensory and microbiological formation, and by up to 30 days when both ethanol emitters and oxygen absorbers were used	[80]
	Iron-based oxygen scavenger sachets	Sachet	Sliced wheat bread	➢ Maintained wheat bread quality for up to 7 days of storage	[81]
	Ethanol emitter	Sachet	Ciabatta bread	➢ Ethanol emitter extended shelf life to 16 days while maintaining acceptable microbiological quality, whereas the usage of ethanol spray revealed no effect on product sensorial properties	[82]
	Oxygen absorber and ethanol emitter	Sachet	Chinese steamed bread	➢ The shelf life of Chinese steamed bread with an oxygen absorber and 1 v% ethanol emitter was extended by up to 11 days	[83]
	Oxygen scavenger and ethanol emitter	Pouch	Sponge cake	➢ The oxygen scavenger and ethanol emitter have high barrier packaging and extended shelf life of sponge cake to at least 42 days by delaying lipid oxidation, color change, cake hardening, and microbial growth	[84]
	Oxygen absorber	Nylon/LLDPE/cast polypropylene film	Preservative-free Chinese pastry (kha-nom pia)	➢ Nylon/CPP film retarded microbial growth better than Nylon/LLDPE and extended shelf life up to 25 days ➢ Hardness of crust and firmness of filling decreased during storage ➢ Oxygen absorber effectively inhibited the growth of total microbial count and yeasts and molds, with no visible mold appearing on the pastries	[85]
	Iron-based oxygen scavenger	Sachet	Preservative-free white bread	➢ The oxygen scavenging sachet’s shelf life lasted for only 4 days ➢ Bread shelf life was prolonged up to 5–7 days with a low initial oxygen level of 5% by volume ➢ When packaging film possesses a high oxygen barrier, an oxygen scavenger is unnecessary	[86]
	Vacuum conditioning	Bag	Chinese steamed bread	➢ Thermal–vacuum packaging kept a higher water content and a longer shelf life, and maintained good taste with lower retrogradation rate of the bread	[87]
	Iron based oxygen absorbers	Bag	Sourdough sliced bread	➢ The most effective application was the high-capacity oxygen absorber combined with 100% N_2_, giving 12 days of a shelf life ➢ With 50% CO_2_ + 50% N_2_, oxygen conc. increased above 2% due to the trapped O_2_ in the pores of bread and had a shelf life of only 3 days ➢ Atmospheric conditions prolonged the shelf life for 6 days	[88]
	Oxygen scavenging compound—pyrogallol	Film	-	➢ Adding the films to the package contributed to lowering oxygen levels in the package headspace for storage at 4, 25, and 50 °C ➢ The maximum oxygen absorption capacity of pyrogallol-incorporated films was 23.0 mL O_2_/g films	[22]
	Ethanol emitter, Oxygen absorber Moisture absorber	Sachet	Refined wheat bread (RWF) and Whole wheat bread (WWF)	➢ Bread packed in a combination of ethanol emitter, oxygen absorber and moisture absorber inhibited growth of microbes effectively. Maximum shelf lives of RWF and WWF were 16 and 8 days, respectively	[89]
	Palladium-based oxygen scavenger	Film	Par-baked bun and toast bread	➢ Scavenger reduced initial oxygen concentration in the headspace from 21% to 2% but was still insufficient to extend the mold-free shelf life ➢ CO_2_ modification in the packaging system extended shelf life to 10–12 days	[90]
	Pyrogallic acid	LDPE/sodium carbonate film	Fish cake	➢ Pyrogallic acid as oxygen scavenging coated on LDPE-based film showed stabilized fish cake quality by improving oxidation properties and inhibiting microbial growth during storage period of 30 days	[91]

## 3. Functional Polymeric Plastic

Polymer materials are a vital part of bakery packaging, as seen in Table 1 and Table 2. They play an important role in protecting food, ensuring freshness and modifying barrier properties such as water vapor and oxygen permeability. Polymer materials also influence mechanical properties of tensile strength and elongation at break, while releasing active compounds which inhibit microorganism growth and extend bakery product shelf life. These polymeric materials can be used to make many product forms including film, tray, rigid container, multilayer film and pouch. The active packaging system can involve non-volatile compounds, volatile compounds, edible mixed polymers, coated polymers, active paper and paperboard, oxygen scavenging, and ethanol emitters (Table 1).

### 3.1. Non-Volatile Active Ingredients

Numerous natural and synthetic ingredients have been incorporated into conventional and biodegradable plastic polymers to produce functional polymers for active packaging. The antimicrobial and antioxidant capacities of these functional polymers depend on several factors, e.g., release behavior, interaction between polymers and ingredients, and morphology of the matrices. Recent applications of these functional polymers are shown in Table 1. To increase the safety and quality of mini panettones, Ferreira et al. (2016) [73] modified citric acid and sorbate potassium by incorporating with edible coating solution, either separately or in combination, to increase shelf life of mini panettone by three times compared to the control. Thanakkasaranee et al. (2018) [38] found that a film made of polypropylene and sodium propionate with a concentration range of 0.5 to 5% enhanced mechanical properties and thermal stability, while increasing hydrophilicity, and demonstrated antimicrobial activity against both Gram-negative and Gram-positive microbes. Packed bread also showed less mold spoilage on day 7 of storage. Tsiraki et al. (2018) [37] investigated a combination of chitosan and natamycin as an effective antifungal agent that delayed the deterioration of phyllo pastry while preserving the basic freshness, look and acceptable sensory properties of the product. Vacuum packing with chitosan and natamycin prolonged the sensory shelf life by 11 days, and microbiological data showed that mesophilic total viable counts, yeasts and molds, psychotropic bacteria, lactic acid bacteria, *Enterobacteriaceae* and enterococci of 1 to 3 log CFU/g on the last day were the most prevalent microorganisms (day 18). Kongkaoroptham et al. (2021) [43] determined that PLA packaging films containing chitosan nanoparticles with polyethylene glycol methyl ether methacrylate (PEGMA) inhibited the growth of natural microorganisms on bread slices. All modified chitosan nanoparticles (CSNPs) showed capacity to inhibit *S. aureus* as high as > 98%, improved elongation at break and oxygen permeation ability in a standard range for food packaging. Sulfur quantum dots (5.3 nm, aqueous suspension) were used by Riahi et al. (2022) [44] in alginate-based multifunctional films for bread packaging. The integrated film revealed tensile strength improvement of 18%, UV barrier property at 82% and antioxidant activity. Film stiffness and water vapor permeability were unaffected. Sulfur-based compounds had antibacterial action against *L. monocytogenes* and *E. coli*, as well as against fungi such as *A. niger* and *P. chrysogenum*. These delayed the appearance of mold on bread for 14 days. The nanosulfur mechanism disrupted metabolic activities by interacting with the target molecules in the microbial cell wall and altering cellular signals. Furthermore, the reactive oxygen species produced by nanosulfur interacted with and weakened the cell walls of microorganisms, causing cell lysis and death. Another mechanism involved the reaction of sulfur nanoparticles inside bacterial cells under acidic conditions, which interfered with cellular component breakdown or prevented DNA replication. Nanosulfur disrupts enzyme SH capabilities that are required for the metabolism of proteins, lipids, and carbohydrates. This results in the breakdown of cellular machinery and cell death [44].

Bio-based polymers, including starch, PBAT, and PLA, showed a high potential to produce biodegradable sustainable packaging [98,99,100]. Likewise, Huntrakul et al. (2020) [101] successfully combined edible heat-sealed acetylated cassava starch with pea protein isolated sachets, demonstrating effective protection for soybean and olive oil stored for up to three months. Pea protein improves interaction between the polymer and glycerol and effectively prevents humidity-induced film shrinkage. To extend the shelf life of bread, Luz et al. (2018) [40] investigated the effects of ε-poly-L-lysine (ε-PL) integrated with a starch-based biofilm as an antifungal agent. They found that ε-PL inhibited growth and showed antifungal efficacy against *A. parasiticus* and *P. expansum*. *A. parasiticus*, the developer of aflatoxin, was also controlled by ε-PL incorporation and diminished aflatoxin by more than 93.90% after 7 days of testing. Sliced bread was packaged in film-forming packaging that contained nanodispersed titanium dioxide (TiO_2_) by Shulga et al. (2021) [42]. Results revealed that 1% TiO_2_ coating increased water vapor barrier properties and inhibited the growth of *Bacillus subtilis* and *Escherichia coli*. Viscusi et al. (2021) [45] studied polypropylene film coated with dispersed anionic clay to host sorbate for white bread packaging. The coated film retained organoleptic characteristics, moisture analysis, peroxide evolution and mold count on the bread for up to 12 days at ambient temperature. Moreover, this active packaging inhibited the growth of *Escherichia coli*, *Pseudomonas aeruginosa*, *Salmonella enterica* subsp. *Arizona*, *Staphylococcus aureus*, and *Campylobacter jejuni*. Braga et al. (2018) [39] combined polyvinyl chloride (PVC) and silver nanoparticles as an active film for bread packaging. The PVC characteristics of the film were enhanced, and 1% Ag-nanoparticles suppressed the growth of microbes in bread stored at 26 °C for 15 days. Diffusion inhibited against *B. subtilis*, *A. niger*, and *F. solani* growth. However, the utilization of nanoparticles for packaging in the food industry requires safety assessments to ensure compliance with regional and global regulations [102].

### 3.2. Volatile Active Ingredients

Volatiles and essential oils are compounds that contribute to characteristic flavors and aromas of food products such as fruits, vegetables, herbs, and spices. These compounds mainly comprise terpenes, alcohols, aldehydes, ketones, terpenoids and apocarotenoids [103]. Natural and synthetic volatile compounds have been incorporated into plastic polymers and used for bakery packaging, as shown in Table 1. Likewise, for white pan bread and butter cake, Klinmalai et al. (2021) [53] noted how this food, when packed in blown-film extrusion of PLA/PBAT integrated with carvacrol essential oils (0, 2 and 5%), showed delayed *Penicillium* sp. and *Rhizopus* sp. growth and sporulation by film containing 2 and 5% carvacrol, with the shelf life extended by up to 4 days. PLA/PBAT blend films with plasticized carvacrol functionalization prevented growth of mold in baked products. Sharma et al. (2022) [57] studied the bacterial-based biopolymer, poly (3-hydroxybutyrate-co-4-hydroxybutyrate) or P(3HB-co-4HB) incorporating thyme essential oil as active packaging for white bread shelf life extension. Shelf life was extended up to 5 days compared with 1–4 days for the neat film, with improved film elongation at break and water vapor permeability. Passarinho et al. (2014) [46] developed an antimicrobial sachet containing oregano essential oil that acted against yeasts, mold, and *Escherichia coli, Salmonella* Enteritidis and *Penicillium* sp. on sliced bread. During storage, γ-terpenes and φ-cymene inhibited yeast and mold growth on bread slices. Ju et al. (2020) [49] discovered that a mixture of essential oils eugenol and citral (1:1) in corn starch microcapsule sachets decreased molds and yeasts from 100% to 56% at 25 °C and from 90% to 26% at 35 °C of storage conditions. Furthermore, the use of essential oils in sachets had minimal effect on the smell or taste of the bread. Sliced bread packed in LDPE, PP and HDPE bags containing the same essential oil sachets did not develop mold until day 16, 14, and 14, respectively. Mahmood et al. (2022) [56] used electrospinning techniques to produce fish-gelatin-based nanofiber mats embedded with cinnamaldehyde (CEO), limonene (LEO), and eugenol (EEO) at 1, 3, and 5% for bread packaging improvement. Results showed that all essential oils had radical scavenging activity such as CEO = 73.50%, LEO = 51.20%, and EEO = 89.37%, which was the highest at 5% concentration, whereas they also showed ferric-reducing antioxidant power and improved encapsulation with the electrospinning method. They also inhibited the growth of *E. coli*, *S. aureus* and *A. niger* because the gelatin-based mats had good release of essential oils, with no fungal spots on bread antimicrobial packing. Balaguer et al. (2013) [25] developed gliadin films incorporating cinnamaldehyde that were highly effective against fungal growth both in vitro and in food packing systems. Cinnamaldehyde volatility from the solution forming film inhibited the activity of *P. expansum* and *A. niger* over 10 days. Similarly, Fasihi et al. (2019) [104] used the Pickering stabilization method to enrich cinnamon essential oil (CEO) and carboxymethyl cellulose (CMC)–polyvinyl alcohol (PVA) in the solution-forming film and bread coating to increase the anti-UV properties and antifungal properties to prolong bread shelf life. Pickering stabilization impacted CEO by several mechanisms including (i) the generation of a uniform and regular structure of dispersed phase throughout the film matrix leading to increased contact between CEO and fungi, (ii) controlled and regular release of CEO from the film to the outside, which maintained sufficient antimicrobial and antioxidant agents in the headspace, and (iii) protection of CEO from oxidation against undesirable external effects that increased its efficiency as an active compound. PLA and PBAT blend films containing *trans*-cinnamaldehyde were studied by Srisa and Harnkarnsujarit (2020) [24]. Results showed increased water vapor and oxygen permeability because blending of PBAT/PLA reduced the orientation and non-homogeneity of the network formation. Volatility was higher at increased cinnamaldehyde concentration, and different blending ratios of the film released compounds and inhibited the growth of *Aspergillus niger* and *Penicillium* sp., effectively inhibiting microorganism growth for up to 21 days at 30 °C with slightly affected organoleptic properties of cinnamaldehyde taint at 5% concentration. Songtipya et al. (2021) [54] designed a patch that combined natural rubber pressure-sensitive adhesive and cinnamon oil for banana cake packaging. The NR-PSA/CO patch delayed the growth of bacterial and fungal strains of *Escherichia coli*, *Staphylococcus aureus* and *Aspergillus niger* with further extension of the 4-day shelf life. Cashew gum and gelatin were combined with ferulic acid and lemon grass essential oil by Oliveira et al. (2020) [52] to develop a casting film that showed increased water vapor permeability, decreased solubility and enhanced mechanical characteristics. The incorporated film also prevented the formation of mold for up to 7 days of storage, but the barrier properties of the film were limited, and bread was harder than commercial packaging (PE). Priyadarshi et al. (2018) [47] produced chitosan (CA) film integrated with apricot kernel essential oil (AKEO) for sliced bread packaging. The blended film increased water vapor barrier performance by up to 41%, with a solubility of only 4.76% and a moisture content of 8.33% compared to the control film of 18.42%, and 16.21%, respectively. This film had enhanced tensile strength and scavenging activity with both H_2_O_2_ and DPPH tests. Moreover, it delayed the bacterial development of *Bacillus subtilis* and *Escherichia coli* and protected sliced bread against fungal growth within the packaging on day 10 with a low concentration ratio of essential oil of 1:0.125 (CA:AKEO) film. Bui et al. (2021) [55] produced a blended film of poly (vinyl alcohol) and *Piper betel* Linn. leaf extract to extend bread shelf life. The film showed high UV blocking and antimicrobial efficiency, with inhibitory efficacy against *E. coli*, *S. typhimurium*, *S. aureus* and *P. aeruginosa* at 3% of extract concentration. Moreover, bread quality was preserved for 45 days at room temperature. Jha (2020) [51] produced bio-nanocomposite films based on corn starch chitosan with plasticizer sorbitol and grapefruit seed extract. The film showed maximum inhibition zone against *A. niger* and extended bread shelf life up to 20 days at 25 ℃ and 59% RH because it had low moisture content, water vapor permeability, solubility, high tensile strength, and high antifungal activity.

Furthermore, based on patents in Table 2, Carolina et al. (2022) [97] found that antifungal packaging comprising a polyolefin with a water-soluble polymer coating such as PVOH with a synergistic mixture of volatile natural compounds selected from carvacrol and allyl-isothiocyanate showed enhanced antifungal activity against the main fungi responsible for damage and spoilage of sliced bread such as *A. niger* and *Penicillium*. Bread samples packed in multilayers and coated with a film of LDPE, EVOH, acrylic coating, and mustard oil as an active essential oil showed improved storage for 30 days without any visible fungal growth on the surface of gluten-free bread [96].

## 4. Functional Paper and Coating Technology

**Figure 3:**
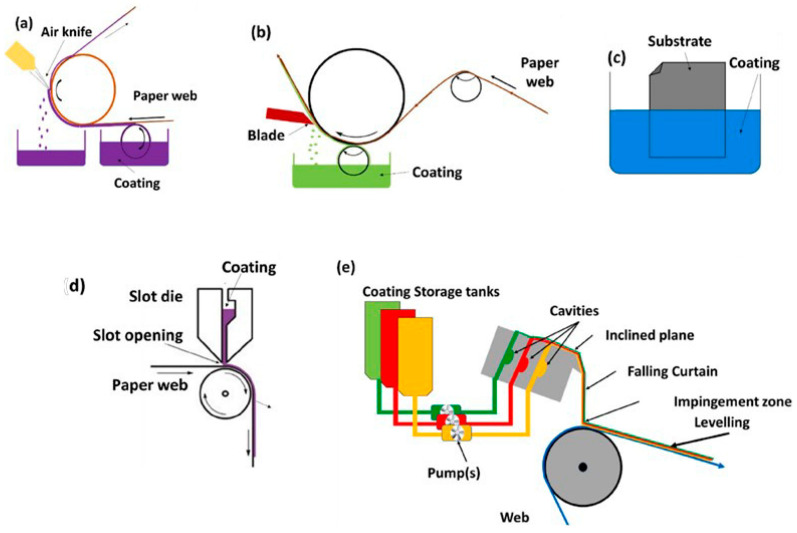
Illustration of different paper coating methods, (**a**) air knife, (**b**) blade metering, (**c**) dipcoating, (**d**) slot die, and (**e**) curtain coating (reproduced with permission from Tyagi et al. (2021) [105]).

Several coating methods as traditional and modern are commonly used for paper packaging. Traditional methods require a size press, rod coater, blade coater, roll-blade coater, and Vari dwell and fountain blade coaters, whereas modern methods employ a dip coater, slot die coater, curtain coater, electrostatic powder coating, spray coater, and lamination with tie layer (Figure 3) [105].

**Figure 4:**
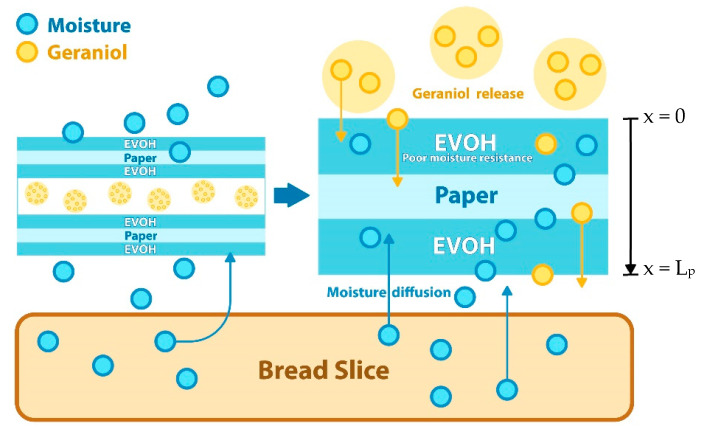
Schematic diagram describing the diffusion mechanism in bread packaging (reproduced with permission from Petchwattana et al. (2021) [70]).

Table 1 lists some recent functional polymers used in paper packaging for bakery products. Numerous coating technologies have been utilized to enhance the functionality of paper packaging. Shankar and Rhim (2018) [67] developed coated wrapping paper using a solution of alginate, carboxymethylcellulose, carrageenan, and grapefruit seed extract. The paper coating resulted in improved mechanical properties, water vapor barrier, surface hydrophobicity, and tensile strength. This antimicrobial paper also inhibited the bacterial growth of *L. monocytogenes* and *E. coli,* with the removal of surface-inoculated bacteria from minced fish cake packaging achieved in 6–9 days. Rodríguez et al. (2008) [62] conducted research on active paper packaging based on the use of solid wax paraffin and cinnamon essential oil. The active coating significantly reduced mold growth by direct migration in packaging and demonstrated resistance to *Rhizopusstolonifer* growth at 4% concentration. Landge et al. (2009) [61] showed that milk cake packed with wax-coated paper pouches demonstrated a high level of sensory evaluation, with an estimated overall acceptability score of 7.2 out of 9.0 and remained acceptable for up to 21 days. Moisture loss from the cake was minimized because the wax coating had high barrier capabilities. Rhim et al. (2007) [59] produced PLA-coated paperboards with improved water barrier properties, decrease in water vapor permeability (WVP), increase in water contact angle (CA), and reduction in water absorptivity (WA) at an optimal concentration of 3% *w*/*v* PLA in chloroform. Antimicrobial sachet systems were designed by Petchwattana et al. (2021) [70] for extending the shelf life of sliced bread by combining paper coated with ethylene vinyl alcohol (EVOH) and poly (butylene succinate) (PBS) with geraniol essential oil (Figure 4). When paper is coated with EVOH, the surface becomes rougher and contains small pores, whereas water vapor transmission rate (WVTR) decreases three-fold, making the paper suitable for use as a release-control component in antimicrobial sachets. Interestingly, the PBS/geraniol-10% pellets exhibited clear zones of inhibition against *Escherichia coli* and *Bacillus cereus* from 5 to 15 mm. The shelf life of the bread in packages containing sachets was also extended compared with uncoated paper since the release of geraniol achieved equilibrium around day 40. The degradation of white bread with total plate count, yeast, and mold count occurred on day 42 when using antimicrobial sachets. No fungus appeared on the white bread surface preserved with an antimicrobial sachet for the entire 63-day test period. The antibacterial function of geraniol in the paper coating suppressed mold formation. The mechanism of the antimicrobial sachet begins with the plasticization of the EVOH material in the presence of increased humidity. This then allows the molecules to pass through the coated paper layers. At the same time, geraniol molecules migrate to the surface of the PBS pellets. When the coated paper swells, the geraniol detaches from the antimicrobial sachet and exerts its antimicrobial functions, with the diffusion value 3.18 × 10^−14^ m^2^/s according to the diffusivity calculation [70].

For the paper packaging of baked products, Rakchoy et al. (2009) [60] coated vanillin with dimethyl sulfoxide, ethyl alcohol, and chitosan at concentrations of 10, 5, 2.5, and 1.125% *w*/*w*. Each coating successfully inhibited the growth of the bacteria; however, the efficiency varied depending on the mixture concentration. Bio-based chitosan-rich starch-coated paper developed by Vrabič Brodnjak (2017) [64] demonstrated more glossiness because the surface pores treated ultrasonically had improved moisture resistance, mechanical characteristics, and flexibility. Apjok et al. (2019) [68] coated cellulose-based papers with chitosan/Ag/TiO_2_ to package clarified butter for six months of storage. After coating with a mixture of chitosan, Ag, and TiO_2_ (P-CH-Ag/TiO_2_), the plain paper had greater opacity, reduced water vapor, and oxygen permeabilities and decreased oil permeability. The coated paper demonstrated good inhibition against *E. coli*, with percentages of 70.36 on an agar plate and 73.28 in butter samples, as well as against yeasts and molds at 77.02% on an agar plate and 79.28% in butter samples. The maximum allowed limit for animal fats is 4 mEq. O_2_/kg; however, after six months, the peroxide value increased 6.47-fold with P-CH-Ag/TiO_2_ compared with uncoated at 36.71-fold [106]. Peter et al. (2016) [63] modified white bread paper packaging with Ag/TiO_2_-SiO_2_, Ag/N-TiO_2_, or Au//TiO_2_ coating. As the coating composite content increased, paper attributes including bursting, tensile strength, tearing, and breaking resistance decreased, whereas paper whiteness increased. By effectively controlling acidity and inhibiting the growth of yeasts and molds, the packaging increased bread shelf life by two days compared with unmodified paper packaging, both in ambient and refrigeration conditions. Mihaly-Cozmuta et al. (2017) [65] developed active cellulose-based papers containing TiO_2_, Ag-TiO_2_, and Ag-TiO_2_-zeolite nanocomposites for bread packaging. The paper integrated with the nanocomposites via oxygen links that improved barrier properties such as air permeance by 4.36-fold (Ag-TiO_2_) and water vapor permeability, whereas permeability to grease decreased by 28.75%. Furthermore, bread packed in Ag-TiO_2_ paper showed increased shelf life at 2 days more than the control package based on yeast and mold growth. Mizielińska et al. (2020) [69] modified a multilayer of paper-based materials with NaCl functioning as a water absorber for bread packing and the relative humidity (RH) of sandwich paper increased to 72%. After 72 h of storage, bread sensory quality and freshness improved, with longer shelf life. Additionally, as shown in Table 2, Sirkku (2010) [94] patented a method for producing coated recyclable paper or paperboard comprised polymer emulsion (acrylic, or styrenebutadiene emulsion) 70–90% dry weight and pigments 10–30% as an aqueous coating layer and then drying and cooling the coated paper or paperboard. Coating improved barrier properties, with water resistance of less than 10 g/m^2^, moisture vapor transfer rate less than 120 g/m^2^, and heat sealability.

## 5. Edible Polymeric Coating in Bakery Products

Edible polymers refer to the polymeric materials that can be easily consumed by humans, animals, or microorganisms without any harmful effects on health. They are categorized into polysaccharides, proteins, and lipids. They have gained increasing application in functional food industries (food packaging and nutrients protection) and biomedical fields (drug delivery, tissue engineering, and wound dressing) [107,108,109]. Edible polymers have been studied and applied as particle form, layer form (coating or film) or as textile structures (edible fibers or modification of non-edible textiles), as shown in Table 1. The coating is a thin layer of liquid applied to the food surface, whereas films are formed on or between the food components and applied as a solid sheet [107,110]. Edible polymers can be made from polysaccharides and proteins and used to preserve quality and extend the shelf life of food. Proteins are composed of amino acids and polysaccharides with long chains of sugar molecules. They can form higher-order structures through a variety of interactions with themselves or with other proteins, including hydrogen and disulfide bonds, London dispersion forces and charge-to-charge interactions. Polysaccharides often have fewer interactions with other polysaccharides, which limits their structural flexibility. These biopolymers have been incorporated with several active ingredients to prolong the shelf life of bakery products. Chitosan is a biopolymer with native antimicrobial functions that have been extensively investigated for active edible coating [111]. For example, in the case, a combination of cassava starch and papain as an active edible film demonstrated successful blending with increased mechanical and surface hydrophobicity characteristics. Papain-blended film also showed its ability to slow the dissolution rate in water [112,113,114]. Lee et al. (2019) [75] used multiple layer films of PP/SAEO/PET/TH/LDPE with star anise essential oil (SAEO) coating the outside that acted as an insect repellent, whereas thymol essential oil (TH) coating the inside functioned as an antimicrobial for sliced wheat bread packaging systems. Results showed that the packaging system sustained insect repellent activity against *P. interpunctella* larvae for up to 23 days and inhibited *S. aureus* and *P. roqueforti* for 14 and 7 days, respectively. These edible layers are used as carriers of active ingredients. Gregirchak et al. (2020) [77] applied an edible coating containing lactic acid bacteria as a probiotic, sodium alginate, whey, and glycerol on wheat bread to protect against microbial spoilage. Results showed that the edible coating of lactic acid bacteria diminished the population of mesophilic aerobic and facultative aerobic bacteria and protected the bread crust against contamination from mycelium fungi of genera *Aspergillus* and *Penicillium*, whereas Altamirano-Fortoul et al., 2012, [71] applied a probiotic coating technique to obtain the microencapsulation of *Lactobacillus acidophilus* and starch-based material coated onto the surface of baked breads. This coating resulted in better protection on bread crust with sensory acceptability. Probiotics do not affect consumer health and are good for the digestive system. Short-term storage caused a reduction in the total colony counts of microencapsulated *L. acidophilus* in all treatments, indicating that a probiotic coating can be applied to protect the bread crust and retain sensory acceptability. Using triticale flour, Bartolozzo et al. (2016) [74] coated muffins with a film-forming solution. The coated muffins had delayed crumb firming kinetics and were softer after 10 days of storage. After 7 days of storage, the coated muffins showed a greater reduction in weight loss than the control sample. Triticale film coating worked well to prolong the staling process, keeping the muffins softer. Okra mucilage was used as the main component of biscuit coating by Senanayake (2021) [78] and showed improved moisture barrier qualities, while maintaining biscuit crispness and reduced deterioration and microbial spoilage. Noshirvani et al. (2017) [36] developed active surface-coating techniques incorporating chitosan, carboxymethyl cellulose, oleic acid, and zinc oxide nanoparticles to prolong sliced bread shelf life by up to 35 days, maintaining moisture content through lower water vapor permeability. Moreover, Ferreira Saraiva et al. (2016) [73] used edible coatings made of potato starch, inverted sugar, and sucrose to control the releasing of both citric acid and sorbate potassium for mini panettone shelf-life preservation.

## 6. Other Forms of Active Packaging Technology for Bakery Products

Some recent patents on polymeric packaging technology for bakery products are shown in Table 2. Most novel investigations aim to improve the quality and freshness of bakery products and also extend the shelf life. Antimicrobial actions of the package have been developed, with package structures engineered to achieve higher quality of packaged bread suitable for commercial production using extrusion processes. Other forms of packaging technology such as oxygen scavengers and ethanol emitters as shown in Table 1.

### 6.1. Oxygen Absorbers

Volatiles and essential oils are compounds that contribute to characteristic flavors and aromas of food products such as fruits, vegetables, herbs, and spices. These compounds mainly comprise terpenes, alcohols, aldehydes, ketones, terpenoids, and apocarotenoids [103]. Packaged foods need to be protected against oxidative deterioration to enhance shelf life and customer acceptability while maintaining food security. Oxygen absorbers play an important role in the removal of dissolved oxygen and preservation of the flavor, texture, and aroma of various food products [115]. Oxygen-scavenging activity strongly depends on package conditions including humidity, temperature, and morphology of the scavengers, which can be in the form of films and sachets [116]. Scott et al. (2006) [93] discovered an absorbent structure comprising PET, indium tin oxide, aluminum, silicone-based chrome complex, and wax materials. The absorbent sheet has a non-stick food-contacting surface and may be incorporated into or used with a tray or formed into a roll of absorbent material comprising at least two overlapping absorbent sheets. Commercial iron-based oxygen scavengers have high efficacy to eliminate residual oxygen in the package headspace to less than 0.01%. Oxygen scavenging activity in iron-based scavengers is catalyzed by humidity and higher at the beginning due to rapid oxidation of reduced iron powders and ferrous iron compounds. The formation of iron rust causes a tortuous path that hinders oxygen diffusion and slows down oxidation, giving lower reaction rates after 24 h [84]. Charles et al. (2004) [92] developed a rigid container that contained an oxygen scavenger and an indicator for bakery packaging. This system had good oxygen barrier properties, measurable oxygen concentration within the headspace of an assembled package, and indicated oxygen levels using a luminescent compound, whereas Muizniece-Brasava et al. (2012) [81] investigated conventional multibarrier pouches (MAP, 60% CO_2_, 40% N_2_) with an embedded oxygen scavenger to maintain wheat bread quality for up to 7 days of storage. Rüegg et al. (2022) [90] developed a catalytic system based on palladium to eliminate oxygen in the headspace and extend the shelf life of bakery products by inhibiting mold growth for 3 to 9 days. Additionally, the combination of PBAT/TPS and TiO_2_ demonstrated effective oxygen-scavenging activity, reducing residual remaining oxygen from the packaging headspace [117]. Upasen and Wattanachai (2018) [86] conducted research on blown films with an LDPE layer combined with oxygen absorbers to extend the shelf life of white bread for 4 days by maintaining fungal and mold development at a lower level than control films, whereas Sheng et al. (2015) [83] used an oxygen absorber and an ethanol emitter together to significantly increase the shelf life of Chinese steamed bread. However, a high ethanol content (3%) produced poor sensory qualities, and the bread was unsuitable for preservation. The shelf life of the sample with an oxygen absorber and 1% ethanol emitter was extended by up to 11 days. Latou et al. (2010) [80] extended the shelf life of sliced wheat bread by up to 24 days using an ethanol emitter and by at least 30 days when both an ethanol emitter and an oxygen absorber were present, whereas Berenzon and Saguy (1998) [79] applied an iron-based oxygen absorber in sachet form to prevent oxidation and extended the shelf life of military ration crackers packaged in hermetically sealed tin cans for 44 weeks. Promsorn and Harnkarnsujarit (2022) [22] combined thermoplastic starch and pyrogallol with LLDPE via cast film extrusion. Films designated with pyrogallol showed lower package headspace oxygen levels. After storage at 4, 25, and 50 °C, the maximum oxygen absorption capacity of pyrogallol-incorporated films was between 2.2 and 7.3, 4.6 and 23.0, and 5.0 and 13.1 mL O_2_/g films, respectively. The investigation of oxygen-absorbing polymers allows wider utilization of oxygen scavengers in the form of non-metallic components. Pyrogallic acid was also used as an oxygen scavenger, and applied as a coating material to the surface of polyethylene film with sodium carbonate (anhydrous). The coated film demonstrated that sodium carbonate accelerated the reaction of pyrogallic acid and oxygen, stabilizing the quality of fish cake by slowing pH reduction and reducing the amount of oxidation. Additionally, PE/SC/PA-20% at 7 log CFU/g suppressed the proliferation of microorganisms after 30 days of storage [91]. Apart from oxygen-scavenging technology, a conventional modified atmosphere which eliminates oxygen levels in the package headspace combined with high oxygen barrier polymeric packaging greatly enhances the shelf life of bakery products. Additionally, Liu et al. (2019) [87] investigated the packaging of Chinese steamed bread such as sealed packaging (SP), vacuum packaging (VP), and thermal–vacuum packaging (T–VP). Results showed that T–VP had higher water content and a longer shelf life because it related to permeability of packaging films which maintained good taste and lower retrogradation rate of the bread because the package delayed the speed of water desorption by blocking the interaction among water molecules, gluten, and starch.

### 6.2. Ethanol Emitters

Ethanol can be sprayed directly on the product or packaging or inside the package using ethanol emitters [118]. According to Dao and Dantigny (2011) [119], the storage of grain and cereal were protected against toxic mold growth via ethanol vapor fumigation in bakery storage room. Dantigny et al. (2005) [120] used ethanol against 12 common food-borne fungi and determined optimal concentration range of 3–5%, whereas Hempel et al. (2013) [82] demonstrated that ethanol emitters increased shelf life without the usage of extra modified environment gas. After utilizing ethanol emitters in package items in air for 16 days, acceptable microbiological quality criteria were maintained. Comparing the usage of ethanol emitters to ethanol spray techniques and controls revealed no difference in the product quality. Moreover, Janjarasskul et al. (2016) [84] also indicated the anti-stalling effect of ethanol emitters that formed ethanol-amylose/amylopectin complexes and reduced retrogradation, whereas ethanol also plasticized protein networks in cake crumb [84,121]. The permeability of the package containing volatile antimicrobial agents can be improved to control the release using composite and laminate materials [122].

## 7. Conclusions and Final Remarks

The incorporating active agents into polymeric materials such as plastic and fibers produces active functional packaging that can preserve the quality and extend the shelf life of bakery products. Several volatile and non-volatile substances have been incorporated into polymers to produce active packaging for bakery products with novel volatile ingredients recently investigated. Other forms of active packaging such as oxygen scavengers and ethanol emitters have been applied for the quality preservation of bakery products. The permeability of polymeric films also plays a key role in quality changes that induce moisture loss and reduce water activity, with subsequent restriction of microbial growth. Challenges for these functional packaging include compatibility between active ingredients and organoleptic quality of the bakery, mass transfers of the active substances from polymeric packaging into foods and levels of migration which should be complied with products legislation. The release of antimicrobial volatile substances has a promising role in delaying mold growth. However, odor and flavor contamination by the incompatible active agents possibly limits their commercial applications. More studies on volatile active compounds which have no effects on organoleptic qualities of bakery products are the future perspective of this area of research. All these relationships should be considered when designing proper packaging to retain organoleptic quality while reducing microbial growth in bakery products.

## Figures and Tables

**Table 2:** Previous recent patents related to packaging technology for bakery products.

Materials and Components	Packaging Form	Package Conversion Technology	Bakery	Key Technology	Results	References
Rigid container Oxygen scavenger and indicator	Oxygen detection system	Rigid container with an oxygen detection system	Bakery products	A rigid container comprising: (a) An oxygen barrier having an oxygen transmission rate of no more than 100 cc/m^2^/24 h at 25 °C, 0% RH, 1 atm; (b) An oxygen scavenger; (c) An oxygen indicator comprising a luminescent compound wherein the oxygen indicator and oxygen scavenger are substantially shielded by oxygen barriers from environmental air.	A rigid container with oxygen barrier properties Can measure oxygen concentration within the headspace of an assembled package Indicates oxygen level with luminescent compound	[92]
PET Indium tin oxide Aluminum Silicone-based Chrome complex Wax	Absorbent sheet	Absorbent structure compression	Bakery products	A structure having absorbent and microwave interactive properties containing: (a) A polymer film: PET, indium tin oxide and aluminum; (b) A layer of microwave energy interactive material: indium tin oxide and aluminum; (c) A liquid-absorbing layer; (d) A liquid-impervious material; (e) A release coating overlying silicone-based material, chrome complex, wax, or any combination thereof	The absorbent sheet had a non-stick food-contacting surface The absorbent sheet can be incorporated into or used with a tray and formed into a roll of absorbent material comprising at least two overlapping absorbent sheets	[93]
Paper or paperboard Polymer emulsion Pigment	Coated paper or paperboard	Paper or paperboard is coated with a polymer emulsion in one or more coating	Bakery products	A method of producing a coated recyclable paper or paperboard comprising: (a) Polymer emulsion (acrylic emulsion, or styrenebutadiene emulsion) 70–90% dry weight, pigment (grade clays, titanium dioxide, calcium carbonate, barium sulfate, talc, zinc sulfate, aluminum sulfate, calcium oxide, lithopone, zinc sulfide, and mixture thereof) 10–30% dry weight; (b) Applying an aqueous coating layer; (c) Drying the coating; (d) Cooling the coated paper or paperboard	Coated paper or paperboard had improved barrier properties including water resistance of less than 10 g/m^2^, moisture vapor transfer rate of less than 120 g/m^2^ and was heat sealable.	[94]
Bimodal ethylene 1-butylene C6–C12-alpha-olefin terpolymer LDPE Metallocene-produced	Multilayer film	Polymerization Coextruded multilayer film	Frozen food packaging Bakery product	The multilayer film comprised a core layer and two outer layers (O-1, O-2) (a) Core layer: bimodal ethylene/1-butylene/C6-C12-alpha-olefin terpolymer (b) Outer layer (O-1): bimodal terpolymer, LDPE, or LLDPE, metallocene-produced (c) Outer layer (O-2): LDPE, or LLDPE, metallocene-produced	The Material had excellent mechanical properties, such as stiffness, toughness, and processability and was suitable for co-extrusion processes	[95]
LDPE EVOH Acrylic coating Mustard oil	Coated film	Multilayers include coating	Gluten-free bread	Antifungal active container comprising a high-barrier co-extruded three-layer film with an outer polymeric layer of LDPE, an intermediate polymeric layer of EVOH and an inner polymeric layer of LDPE which carried or incorporated mustard oil	Bread samples lasted for 30 days without any fungal growth visible on the surface, whereas the control samples developed a bad taste due to retrogradation of starch	[96]
Polyolefin LDPE PVOH Carvacrol Allyl-isothiocyanate (AITC)	Film	Coating film	Sliced bread	Antifungal packaging comprising a polyolefin with a water-soluble polymer coating as a synergistic mixture of volatile natural compounds selected from carvacrol and allyl-isothiocyanate for extending the shelf life of bakery products	PVOH had sufficient coating functional properties, showing high uniformity and adhesion to PE, whereas the combination of carvacrol and AITC showed enhanced antifungal activity against the main fungi responsible for damage and spoilage of sliced bread: Aspergillus niger and Penicillium	[97]

## Data Availability

The data presented in this study are available on request from the corresponding author.
